# Radiographic Impact on the Clinical Decision Making of Achilles Tenotomy in Clubfoot: In Search of an Objective Cut-Off Value

**DOI:** 10.3390/jcm13030714

**Published:** 2024-01-26

**Authors:** Mehmet Burak Yalçın, Ahmet Dogan, Onat Uzumcugil, Gazi Zorer

**Affiliations:** 1 Department of Orthopedics and Traumatology, Bahcelievler Memorial Hospital, Istanbul 34180, Turkey; 2 Independent Researcher, Istanbul 34158, Turkey; orthopaedy@hotmail.com; 3 Independent Researcher, Istanbul 34158, Turkey; onat.dr@gmail.com; 4 Independent Researcher, Istanbul 34158, Turkey; gzorer@gazizorer.com

**Keywords:** Achilles tenotomy, clubfoot, Ponseti, radiology

## Abstract

Background: We tried to determine whether the indication of Achilles tenotomy (AT) in clubfoot patients based on clinical evaluation could be confirmed radiographically, and to find an objective radiographic cut-off value for its indication. Methods: Eighty-six clubfeet from 60 patients, (26 bilateral and 34 unilateral) were included. A standard Ponseti treatment regimen was applied. Group 1 comprised patients who underwent AT immediately after serial plaster casting (26 feet). Group 2 comprised patients who underwent AT during the follow-up period (48 feet). Group 3 comprised patients who were assumed to have a corrected foot and did not undergo AT (12 feet). Group 4 comprised the healthy sides of the unilateral cases (34 feet). Results: Both Group 1 and Group 2 showed significant improvement after tenotomy (*p* = 0.002). In order to differentiate between the normal and AT groups according to the pre-tenotomy angle, we obtained an optimal cut-off value of >85° according to the Youden index, a sensitivity of 96%, a specificity of 91.2%, a positive predictive value of 95.9%, a negative predictive value of 91.2%, and an accuracy rate of 94.4% (AUC: 0.983; *p* < 0.001). Conclusions: Feet with a lateral tibio-calcaneal angle > 85° can be considered pathologic and accepted as candidates for AT.

## 1. Introduction

Today, there is nearly a universal consensus that the initial treatment of idiopathic congenital clubfoot should be non-operative and/or minimally invasive, regardless of the severity of the deformity [1,2,3,4,5]. Ponseti [6] demonstrated that the correction of most clubfeet in infants is possible when using proper manipulative techniques followed by the application of well-molded long-leg plaster casts and a percutaneous Achilles tenotomy (PAT) to correct residual equinus contracture.

In the literature, the indication for PAT varies between 41% and 97% [2,7] but it is commonly accepted to be necessary in 80 to 85% [1,6,8,9] of cases. In his original technique, Ponseti utilized clinical evaluation for the tenotomy decision and he did not recommend the standard use of radiographs. An ankle dorsiflexion angle of <15° was recommended as a cut-off value for the performance of PAT [6].

The criteria to perform PAT are based on clinical examination which, in our opinion, is not very clear and subjective. In the literature, the fact that indications for PAT vary between 41% and 97% [2,7] suggests the necessity for an objective criterion. The recent literature contains several studies in this regard [5,7,10,11,12,13]. Ideally, clubfoot treatment should be performed by pediatric orthopedists but, when considered globally, general orthopedists undertake this treatment. This may not be a problem for an experienced physician who is specifically interested in pediatric orthopedics since he/she can visualize the location of the foot bones and the degree of alignment or incompatibility of the joints in three dimensions as if he/she is looking at a photograph of a dissected foot. However, in infants, excess subcutaneous fat tissue or mild iatrogenic rocker bottom deformity due to pseudo-correction originating from midfoot breakage may mislead the surgeon into thinking that the foot is corrected, and such a pseudo-correction can lead to under-treatment. When the equinus has not been fully corrected, we expect a mild recurrence with the loss of dorsiflexion, leading to problems such as wearing orthoses, a subsequent decline in compliance with orthotic use, and further relapse [11]. Clinical evaluation of a complex three-dimensional foot deformity such as clubfoot is relatively difficult and requires a learning curve. Many orthopedic surgeons still rely on radiographs to assess the correction before or after PAT and to detect subtle midfoot changes that would point to the development of a rocker-bottom deformity [10,11].

Since 2003, we have always checked the lateral radiographs with the foot and ankle in neutral dorsiflexion (90°) immediately after Ponseti serial cast treatment (pre-PAT) and right after the final cast removal (post-PAT) in clubfoot. In all cases, the decision for an achillotomy was given by the senior author with clinical evaluation as in the original technique, regardless of radiographic evaluation. In this study, we tried to obtain a radiographic confirmation of the intra-observer clinical evaluation and sought an answer to the question of whether an objective radiographic cut-off value for Achilles tenotomy could be determined.

## 2. Materials and Methods

After obtaining approval for this study protocol from the institutional review board of our institution, we retrospectively reviewed the records of patients who had been treated for clubfoot between March 2003 and April 2019. The inclusion criteria were diagnosis of idiopathic clubfoot treated by the classic Ponseti serial casting method in the first 4 months of age and a minimum follow-up period of 18 months. Non-interrupted treatment, regular follow-up intervals, and accessibility of medical records were also necessary for inclusion. To sustain homogeneity, patients with non-idiopathic (syndromic or neuromuscular) clubfeet were excluded from the study. A total of 82 patients with 124 feet were presented and treated with the Ponseti serial casting method. Only 86 feet from 60 idiopathic patients (42 males, 18 females) were included in our study. The clubfoot deformity was bilateral in 26 patients (52 feet) and unilateral in the remaining 34 patients (34 feet). We strictly applied the Ponseti treatment regimen, as originally described by Ponseti [6].

The mean age of the patients at the time of the first cast application was 12.6 days (range: 1 to 102 days). In all cases, a plaster cast was changed on a weekly basis (serial casting in all cases was carried out by an experienced pediatric orthopedic surgeon), and as a result of the clinical evaluation (a thorough clinical inspection and palpation, an empty heel pad and a high-rising posterior tuberosity of the calcaneus, and <15° ankle dorsiflexion), PAT was performed on those who decided to undergo achillotomy. Prior to tenotomy, full correction of the cavus, hindfoot varus, forefoot adduction, and inversion deformities with the use of casts was clinically confirmed. To prevent iatrogenic rocker-bottom deformities, the surgeon did not try to fully correct the ankle equinus deformities.

Four study groups were formed. Group 1 comprised patients who underwent Achilles tenotomy immediately after serial plaster casting (26 feet of 18 patients). Group 2 comprised patients who later underwent tenotomy during the follow-up period (48 feet of 32 patients). Group 3 comprised patients who were assumed to have a fully corrected foot and did not undergo tenotomy (12 feet of 10 patients). Group 4 comprised the healthy sides of the unilateral cases (34 feet of 60 patients). The decision for tenotomy in Group 2 was given through clinical examinations during the periodic follow-ups.

Percutaneous Achilles tenotomy was performed in the operating room with the patient under general anesthesia. Following the PAT, a long leg cast was applied with the ankle in maximal dorsiflexion and 60° to 70° of abduction, and the foot rotated outwardly in relation to the thigh. This cast was worn for an additional three weeks. No intra-operative or post-operative complications were recorded.

The mean age of the patients at the time of PAT was 8.3 weeks (range: 5 to 18.6 weeks) in Group 1 and 23.8 weeks (range: 17.7 to 49.4 weeks) in Group 2. The patients were followed up for an average period of 71 months (range: 19 to 153 months).

**Figure 1:**
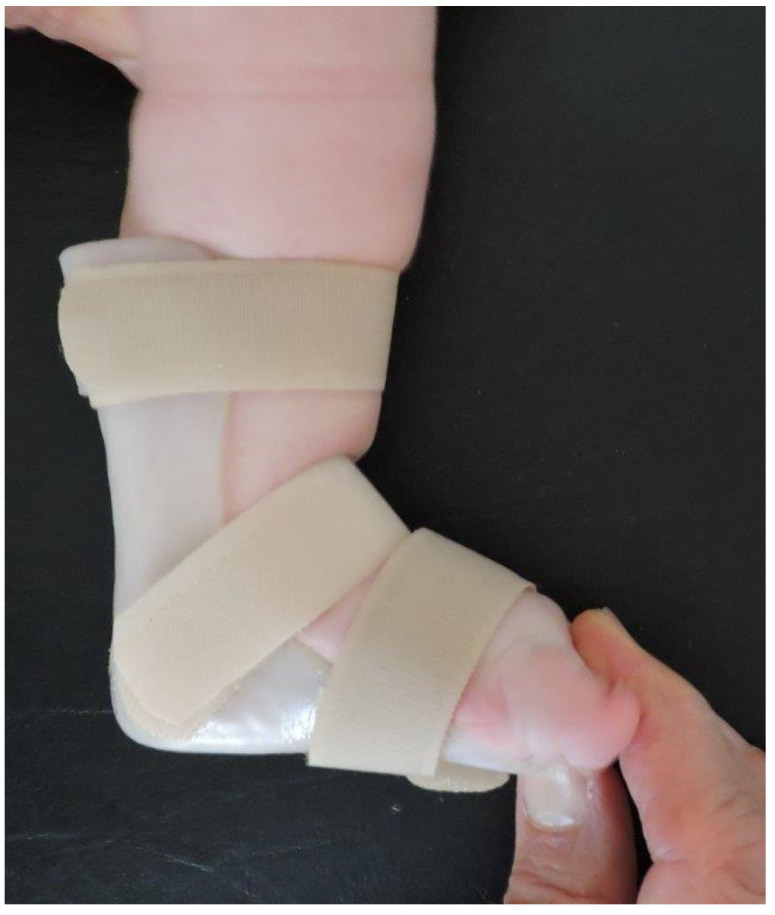
Ankle-foot orthosis that we use in the treatment of our patients.

In both the tenotomy and non-tenotomy groups, after the final cast was removed, we recommended the full-time use of an ankle-foot orthosis (Figure 1) (except for those given five to six sets of passive ROM exercises every 10 min/day), not a bar-connected brace (Denis Browne abduction orthosis), for all patients. All patients were scheduled for regular follow-ups at the outpatient clinic to check for possible relapse deformities.

### 2.1. Radiographic Evaluation

**Figure 2:**
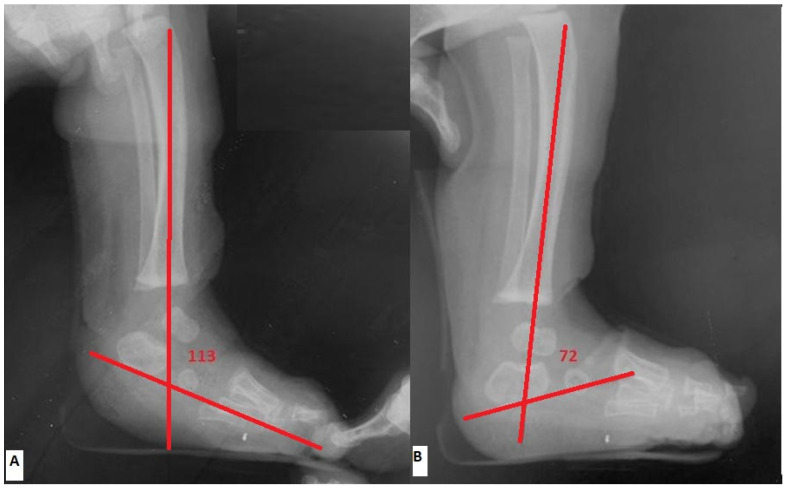
Radiographic evaluation by assessing the lateral tibio-calcaneal (TibC) angle on true lateral radiographs. (**A**) pre-operatively (**B**) post-operatively.

Radiographic evaluation was made pre-operatively (within 4 days prior to tenotomy) and post-operatively (within 7 days right after the final cast removal) by assessing the lateral tibio-calcaneal (TibC) angle on true lateral radiographs, as depicted in Figure 2. The lateral TibC angle was measured based on the angle formed by the intersection of a line drawn through the longitudinal axis of the tibia and a line drawn through the long axis of the calcaneus [3,14,15]. In cases where the tangent of the bottom edge of the calcaneus nucleus was uncertain, the long axis of the ossific nucleus was used as a reference.

In order to obtain standardization and eliminate projection errors, both pre-operative and post-operative radiographs were taken under the senior author’s direct supervision, with the foot placed in a rigid plastic ankle foot orthosis (AFO) at 90° in all cases. In infants, weight-bearing was simulated in AFO in order to simulate the normal physiological position (inclination/declination) of the calcaneus while the patient was in a standing position. In simulated standing, the position of the TibC angle is a measure or reflection of calcaneal alignment. Therefore, radiographs were taken with the foot in neutral dorsiflexion (i.e., with the child’s foot in a neutral position at the ankle joint).

### 2.2. Statistical Analysis

Statistical analysis of the variables was performed with the SPSS v.25.0 (IBM Corp., Armonk, NY, USA) and MedCalc 14 (MedCalc Software Ltd., Ostend, Belgium) software. While conformity of the univariate data with normal distribution was checked with the Shapiro–Francia test, Levene’s test was used for variance homogeneity. In a comparison of two repeated measurements of dependent quantitative variables with each other, a paired-samples t-test was used with Bootstrap results. In order to confirm the relationship between the classification of the groups based on the cut-off value calculated according to the variables and their actual classification, sensitivity, specificity, positive predictive value, negative predictive value, and accuracy rate were analyzed and expressed with a ROC (receiver operating characteristic) curve. Quantitative variables were expressed as mean ± SD in the tables, while categorical variables were shown as n (%). Variables were examined at a 95% confidence level, and a *p*-value of less than 0.05 was considered statistically significant.

## 3. Results

**Figure 3:**
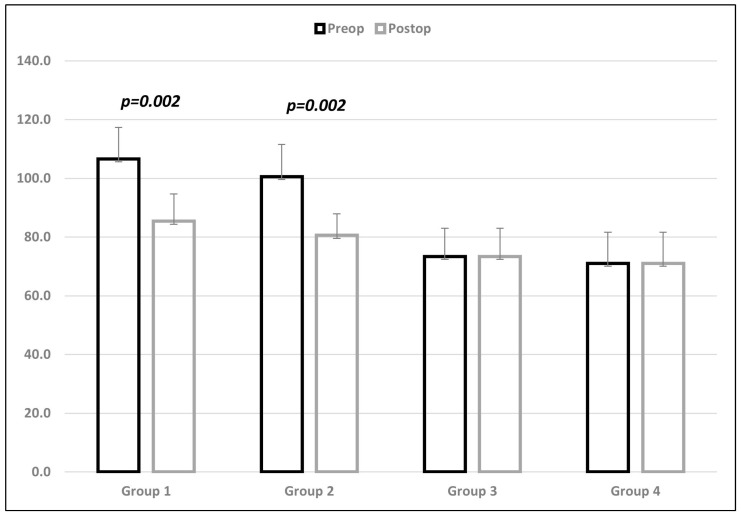
Tibio-calcaneal angle value distribution and standard deviation in each group, values in degrees.

Percutaneous Achilles tenotomy was required in 74 (86%) of the 86 feet. The average and standard deviation values of the angular parameters for each group are given in Table 1. The lateral TibC angle declined 21.2° (range: 16° to 26°) in Group 1 and 20° (range: 15° to 23°) in Group 2. The improvement after PAT was significant in both groups (*p* = 0.002) (Figure 3). There was no significant difference between Group 1 and Group 2 between pre-PAT (*p* = 0.101) and post-PAT (*p* = 0.131) angular values. There was no significant difference between Group 3 and Group 4 in terms of angular measurements (*p* = 0.913). However, a significant difference was observed between Group 1 and Group 3 (*p* < 0.001), Group 1 and Group 4 (*p* < 0.001), Group 2 and Group 3 (*p* < 0.001), and Group 2 and Group 4 (*p* < 0.001) before and after PAT.

Twenty-six cases in the PAT group (Group 1 + Group 2) and eight cases in the non-PAT group (Group 3) had unilateral involvement (Table 2). Both pre-PAT (*p* < 0.001) and post-PAT (*p* = 0.002) angular values showed a significant difference in comparison to the PAT group of the normal sides. The difference in terms of angular values was not significant between the non-PAT group and the normal sides (*p* = 0.126).

In order to differentiate between the normal group (Group 4) and the PAT groups (Group 1 + Group 2) according to the pre-PAT angle variable, we obtained an optimal cut-off value of >85° (CI: >82°–>92°) according to the Youden index, and a sensitivity of 96% (CI: 88.6–99.2%), specificity of 91.2% (CI: 76.3–98.1%), positive predictive value of 95.9% (CI: 88.6–99.2%), negative predictive value of 91.2% (CI: 76.3–98.1%), and an accuracy rate of 94.4% (CI: 84.7–98.9%), while the area under the ROC curve was 0.983 ± 0.009. The success of differentiating the group with a normal cut-off value from the group with PAT was statistically significant (*p* < 0.001). A detailed analysis is given in Table 3.

In pre-PAT evaluations, the TibC angle was above the determined cut-off value in all cases in Group 1. In Group 2, the angle value was below the cut-off value in three cases, (78°, 82°, and 84°). In Group 3, two cases had a TibC angle above the cut-off value (86° and 86°). Three cases in Group 4 had a TibC angle above the cut-off value (90°, 90°, and 92°).

Post-PAT evaluations revealed that 14 cases in Group 1 still had a TibC angle above the determined cut-off value (range: 86°–100°). In Group 2, the TibC angle in 12 cases was above the cut-off level (range: 86°–96°).

## 4. Discussion

Although there are numerous clinical and radiographic methods published regarding the evaluation of clubfoot patients both before and after treatment, there is still no fully agreed and standardized method [10,12,13,14]. Since the clubfoot deformity is a complex, three-dimensional, and partially dynamic one, its classification is challenging and requires a learning curve. The ideal method should be objective and reliable, developable, clinically practical, and guidable in terms of treatment course and results. From this perspective, the two most widely accepted methods today are the Dimeglio and Pirani scoring systems, which have provided the use of a relatively common language. These scoring systems are often used to monitor the treatment process and predict the need for tenotomy, the number of plaster casts, and the recurrence rates [3,14]. However, since physical examination lacks objectivity and reliability for determining the true need for percutaneous Achilles tenotomy, a more objective measurement is needed.

Percutaneous Achilles tenotomy has generally been indicated for feet with an ADF angle of <15° as measured upon physical examination [6]. However, such a physical measurement is often clinically difficult and inaccurate since the infant’s clubfoot is very small and has a possibility of pseudo-correction originating from midfoot breakage. In their study, Radler et al. [11] mentioned that seven feet had good clinical dorsiflexion despite a high lateral TibC angle. Partial pseudo-correction, or a mild rocker-bottom deformity, with dorsiflexion in the midfoot, was noted on the lateral radiographs of these feet, and a PAT was performed despite the measurement of a dorsiflexion greater than 15°. As a matter of fact, this is exactly the problem in this issue; you cannot always reach the right result with just clinical evaluation. In addition, according to the literature, although PAT is decided in 80% of the evaluated cases on average [6,13], different publications have reported a broad range between 41% and 97% [2,7]. This wide range actually indicates the need for an objective criterion for decisions on PAT. In our study, the indication for PAT was primarily determined by the senior author through clinical evaluation, and this decision was confirmed and standardized using radiographic evaluation.

Ponseti [6] did not recommend the standard use of radiographs; instead, he suggested that palpation alone should be employed to assess correction. He mentioned that the evaluation of the actual state of the tarsal bones during early infancy could only be made relatively since the ossification centers of the three visible tarsal bones (talus, calcaneus, and cuboid) are small, still have a more or less circular ossific nucleus, and are located eccentrically. However, a later study of MR images of an older pediatric age group showed a similar orientation of the cartilage anlage and the ossific nucleus [15]. Goksan et al. also mentioned that radiographic abnormalities are common in clubfoot patients [4]. On the contrary, Radler et al. demonstrated that the inter-observer reliability of interpreting radiographs was higher than expected in connection with pediatric feet, especially with increasing age [16]. In another study, it was reported that reproducible radiographic measurements can be made using forced lateral radiographs in infants with clubfeet treated with the Ponseti method by readers of varying levels of experience before and after PAT [3]. Thometz et al. examined the inter-observer and intra-observer reliability of six orthopedic residents after measuring different standard radiologic angles of 30 clubfeet at two separate readings and found that radiographic evaluation was sufficiently reproducible [17]. Radiographic measurements allow us to objectively evaluate the outcomes of foot disorders, including clubfoot [9,10,18].

Radler et al. evaluated all angles in pediatric feet in a radiographic inter-observer reliability study on infants aged 3–12 months (which is the age group in which Achilles tenotomy was most common) and showed that the lateral TibC angle had the highest accuracy in almost all cases (ICC: 0.9758) [16]. In their study of the reliability of radiographic measures in infants with clubfoot treated with the Ponseti method, Zimmerman et al. reported the intra-reader ICC value for the lateral TibC angle as 0.994 and the inter-reader ICC value as 0.910 [3]. Gheldere and Dacquier reported the intra-observer error for the lateral TibC angle as 0.43 degrees [2]. Noh and Park also mentioned that the most predictive factor in their study was the lateral TibC angle, for which the axes were drawn in a constant and obvious line, and the measurements were considered to be sufficiently reliable [5]. Mishima et al. mentioned that the lateral TibC angle demonstrated a significant prognostic factor for rigid relapse in their study [12]. Since this angle is easy to evaluate and has a very high accuracy rate, we wanted to proceed with this angle in our study.

In our study, only the lateral TibC angle was used as a radiographic parameter since it analyses the relation between the tibia and the calcaneus. The main structure between them is the Achilles tendon, which is inserted at the posterior tuberosity of the calcaneus. This angle should be highly influenced by PAT. There is no direct relation between the Achilles tendon and the talus bone. Some publications in the literature have shown that the talo-calcaneal angle of the hindfoot is significantly affected after tenotomy [2,9], while others have reported otherwise [11]. In other words, the position of the talus is not much affected by tenotomy, so the basic determinant of the hindfoot equinus is the position of the calcaneus relative to the tibia. Although Li et al. indicated that the lateral talo-calcaneal angle reflected the relative position of the talus and calcaneus and therefore could be considered an indicator of subtalar joint alignment and motion [9], the clinical reflection of the abnormality at the talo-calcaneal angle on the sagittal plane is uncertain.

In numerous studies, the lateral TibC angle during maximum dorsiflexion on lateral radiographs at maximum dorsiflexion has been used as an evaluation criterion [2,3,7,10,11]. In our study, we used lateral radiographs taken during neutral dorsiflexion instead of maximum dorsiflexion. We found it more appropriate to evaluate the physiological position of the calcaneus when the ankle was flexed at 90°, because the Achilles tendon is mainly, if not solely, responsible for the calcaneal equinus. The TibC angle is a reflection of calcaneal alignment. Therefore, the TibC angle was evaluated using the lateral radiographs taken while the ankle was held in a neutral position with regard to the ankle joint (i.e., a simulated standing radiograph). We believe that the foot being in maximum dorsiflexion or in the neutral position does not have an effect on the radiographic evaluation technique when measuring the lateral TibC angle.

Many authors have reported that a clinical outcome cannot be predicted from radiographs; instead, maintenance of good movement is crucial to a successful outcome whether or not some deformity persists because movement has been shown to correlate with good functional results [4,16]. Decisions made with radiographic examination have been shown to provide a significant improvement in the lateral TibC angle in many studies [3,10,11]. In our study, the desired improvement after PAT was statistically significant, a finding consistent with the literature. From a clinical perspective, this means the gain of ankle dorsiflexion in the sagittal plane. However, in clinical practice, satisfactory dorsiflexion gain after PAT may not always be achieved. This is a clinical observation and radiographic evaluation can also be an indicator of whether a complete tenotomy had been performed in this case. In the literature, it has been indicated that the changes in the lateral TibC angle after PAT are essentially the same as the changes in dorsiflexion as measured clinically [10,11]. In fact, their outcomes can be interpreted as a radiographic confirmation of clinical evaluation. Therefore, a good functional result in the sagittal plane and a good dorsiflexion is directly related to the calcaneal alignment which can be evaluated with the lateral TibC angle.

The most crucial result of our study is the cut-off value for the TibC angle (>85°), which we obtained in regard to the pre-PAT angle variable of the PAT group and the group we assumed to have a normal angle value. Using this cut-off value, we obtained a sensitivity of 96%, specificity of 91.2%, positive predictive value of 95.9%, negative predictive value of 91.2%, and an accuracy rate of 94.4%, while the area under the ROC curve was found to be 0.983 ± 0.009. The success of this cut-off value in differentiating the group with a normal cut-off value from the group that underwent PAT was statistically significant (*p* < 0.001).

In their study, Noh and Park [5] mentioned that they have regarded a forced lateral TibC angle of >80° to be an indicator of PAT and concluded that clubfeet patients with an increased lateral TibC angle and a reduced talo-calcaneal angle prior to PAT possessed a higher risk of residual equinovarus deformities than those with a reduced lateral TibC angle and an increased talo-calcaneal angle.

However, the authors did not provide an explanation as to how they determined the 80° cut-off value. In addition, Kang and Park conducted a similar study in which they considered a lateral TibC angle >80° on the maximal ankle-dorsiflexion lateral view to be an indicator of the performance of PAT [10]. The authors categorized the patients by evaluating the lateral TibC angle and ankle dorsiflexion together, but similarly, they did not provide any explanation as to how they determined the 80° for the cut-off value. It seems that the cut-off value of 80° in both studies was determined blindly and evaluations were made on this basis. In clubfoot deformity, the hindfoot is always in the equinus position, and since the lateral TibC angle is over 90° in most cases, a radiograph taken in neutral dorsiflexion may be interpreted as if it was taken in maximal dorsiflexion. However, in some cases, a decrease of 5 to 10 degrees in the lateral TibC angle may be achieved, thus it can be said that our cut-off values are almost identical.

There was no significant difference between the feet in the group that was switched to orthosis treatment (without tenotomy) after serial casting, assuming that correction of the feet was achieved, and the normal feet in the whole population. This is a finding that supports our result upon clinical evaluation. Similarly, the fact that there was no significant difference between the group for which the PAT decision was made immediately after serial casting and the group for which the PAT decision was taken in the follow-up period, both in the pre-PAT and post-PAT periods, also supports our clinical evaluation result. Therefore, we believe that there is no need to expedite the tenotomy in borderline cases with a PAT indication. According to our study, performing the PAT during the follow-up period does not have a negative effect on the radiographic correction degree and interval. Radler et al. also reported that early Achilles tenotomy does not produce a significant difference in terms of the degree of correction; however, they suggested that it reduces the need for more extensive surgery or the rate of recurrence [19].

Based on the average values of the lateral TibC angle, the significant difference between the PAT feet and non-PAT and normal feet shows that the clubfoot feet were still slightly in the equinus position. This result may be a clue for possible future relapse, but this is the subject of a separate study that requires follow-up. With PAT, an average dorsiflexion gain of 15° to 20° can be achieved [13]; therefore, a complete recovery should not be expected in patients with severe equinus. In addition, we rightly performed PAT in this age group since we did not have any other treatment option other than follow-up with orthoses.

This paper had some limitations. The cut-off value we determined with this narrow group study should be supported by larger study groups and multiple studies. The use of AFO instead of bar-connected abduction orthosis in our patients is different from the universal application and is the personal preference of the authors. However, the use of AFO is not a factor affecting the result of the study as it comes into play after the study subject. Its effect in terms of relapse may be the subject of a separate study. Attending the radiography shots at all times, as in our study, may not be always possible. However, you do not need highly experienced radiology technicians to perform these studies accurately because the equinus is a single-plane deformity (as we accept that other deformity components have been corrected in the pre-PAT period), giving a fully lateral position to a foot placed in AFO should be easily performed by any radiology technician. Unlike other studies in the literature, our failure to take the radiographs while the foot was in maximum dorsiflexion may seem to be another limitation. However, as mentioned above, we aimed to evaluate the physiological alignment of the calcaneus in the simulated standing position. We are of the opinion that taking radiographs at maximum dorsiflexion or neutral dorsiflexion does not change the reproducibility of the results. We agree that the use of radiography is a disadvantage, but as stated above, the radiation dose given to the patients was not a serious dose [20,21]. In addition, we do not recommend the standard use of multiple radiographs in every step during the treatment of clubfeet with the Ponseti method; a single lateral foot radiograph is sufficient to make the decision of Achilles tenotomy at the end of the serial casting application. Thus, we believe that the benefits of the additional information provided by the radiographic assessment of clubfeet outweigh the risk of radiation exposure.

## 5. Conclusions

In conclusion, our clinical evaluation which led us to decide the indication for PAT was confirmed radiographically. The objective radiographic cut-off value we reached as a result of this study is helpful in certain cases to determine the need for a PAT and to detect pseudo-correction, which can be treated with a simple tenotomy, to ensure sufficient correction in the learning curve period and also in unusual cases. Feet with a lateral TibC angle >85° on the radiographs taken via the above-mentioned method can be accepted as pathologic and also a candidate for PAT. In addition, there is no need to expedite the tenotomy in borderline cases with a PAT indication since, based on our study outcomes, performing the PAT during the follow-up period does not have a negative effect on the radiographic outcome.

## Figures and Tables

**Table 1:** Mean values of the tibio-calcaneal angle (in degrees), with the standard deviation for each variable measured in the patients. The values in Group 3 and Group 4 were given the same values because tenotomy was not performed. (*p* < 0.05 significant, SD: Standard deviation).

	Group 1 (N = 26)	Group 2 (N = 48)	Group 3 (N = 12)	Group 4 (N = 34)
	Mean ± SD	Mean ± SD	Mean ± SD	Mean ± SD
Before tenotomy	106.6 ± 10.8	100.6 ± 11.0	73.4 ± 9.6	71.1 ± 10.5
After tenotomy	85.4 ± 9.3	80.6 ± 7.3	73.4 ± 9.6	71.1 ± 10.5
*p* value	0.002	0.002	-	-

Paired-Samples *t*-test (Bootstrap), SD: Standard Deviation.

**Table 2:** Comparison of unilaterally operated patients with their normal feet. The values in Group 3 were given the same values because tenotomy was not performed.

	Group 1 + 2	Group 1	Group 2	Group 3
	(n = 26)	(n = 10)	(n = 16)	(n = 8)
	Mean ± SD	Mean ± SD	Mean ± SD	Mean ± SD
Pre-tenotomy	100.1 ± 12.1	105.9 ± 12.9	96.5 ± 10.4	70.3 ± 9.3
Normal	72.6 ± 10.5	70.6 ± 8.6	73.9 ± 11.6	66.0 ± 9.4
*p* Value	<0.001	<0.001	<0.001	0.126
Post-tenotomy	82.7 ± 8.1	83.2 ± 8.9	82.4 ± 7.8	70.3 ± 9.3
Normal	72.6 ± 10.5	70.6 ± 8.6	73.9 ± 11.6	66.0 ± 9.4
*p* Value	0.002	0.010	0.049	0.126

Paired-Samples *t*-test (Bootstrap), SD: Standard Deviation.

**Table 3:** Univariable analysis for the rigid relapse in each of the ten radiographic measurements. Independent risk factors for the rigid relapse in the multivariable analysis.

	Cut Off (CI) *	Sensitivity (CI)	Specificity (CI)	PPV (CI)	NPV (CI)	AUC ± SE.	*p* Value
For pre-tenotomy angle values							
Group 4 → Group 1 + 2	>85 (>82 to >92) *	96.0 (88.6–99.2)	91.2 (76.3–98.1)	95.9 (88.6–99.2)	91.2 (76.3–98.1)	0.983 ± 0.009	<0.001
Group 4 → Group 1	>92 (>85 to >92) *	92.3 (74.9–99.1)	100.0 (89.7–100.0)	100.0 (85.8–100.0)	94.4 (81.3–99.3)	0.994 ± 0.006	<0.001
Group 4 → Group 2	>85 (>82 to >92) *	93.8 (82.8–98.7)	91.2 (76.3–98.1)	93.7 (82.8–98.7)	91.2 (76.3–98.1)	0.976 ± 0.013	<0.001
Group 4 → Group 3	>61 (>58 to >70) *	100 (73.5–100)	20.59 (88.7–37.9)	30.8 (17.0–47.6)	100.0 (59.0–100.0)	0.565 ± 0.095	0.494

ROC (Receiver Operating Curve), Analysis (Honley and McNell—Youden index J), AUC: Area under the ROC curve, SE: Standard Error, CI: Confidence intervall, PPV: Positive predictive value, NPV: Negative predictive value, * Bootstrap confidence interval (1000 iterations; random number seed: 978).

## Data Availability

Dataset available on request from the authors. The raw data supporting the conclusions of this article will be made available by the authors on request.
